# Phase separation of low-complexity domains in cellular function and disease

**DOI:** 10.1038/s12276-022-00857-2

**Published:** 2022-09-29

**Authors:** Jiwon Lee, Hana Cho, Ilmin Kwon

**Affiliations:** 1 Department of Anatomy and Cell Biology, Sungkyunkwan University School of Medicine, Suwon, 16419 Korea; 2 Department of Physiology, Sungkyunkwan University School of Medicine, Suwon, 16419 Korea

**Keywords:** Molecular biology, Biochemistry

## Abstract

In this review, we discuss the ways in which recent studies of low-complexity (LC) domains have challenged our understanding of the mechanisms underlying cellular organization. LC sequences, long believed to function in the absence of a molecular structure, are abundant in the proteomes of all eukaryotic organisms. Over the past decade, the phase separation of LC domains has emerged as a fundamental mechanism driving dynamic multivalent interactions of many cellular processes. We review the key evidence showing the role of phase separation of individual proteins in organizing cellular assemblies and facilitating biological function while implicating the dynamics of phase separation as a key to biological validity and functional utility. We also highlight the evidence showing that pathogenic LC proteins alter various phase separation-dependent interactions to elicit debilitating human diseases, including cancer and neurodegenerative diseases. Progress in understanding the biology of phase separation may offer useful hints toward possible therapeutic interventions to combat the toxicity of pathogenic proteins.

## Cell biology: creating order from chaos

A physical process known as phase separation allows seemingly chaotic protein sequences to organize into functional structures that can perform important biological functions in both health and disease states. Many proteins naturally fold into well-defined three-dimensional shapes, but others consist of ‘low-complexity’ sequences that contain a limited number of amino acids and lack clearly defined structures. Researchers led by Hana Cho and Ilmin Kwon at Sungkyunkwan University, Suwon, Korea, have reviewed how chemical or physiological conditions trigger the phase separation process which causes these sequences to condense into labile yet functional structures. They explore how reversible phase separation can contribute to the dynamics of intracellular spatial organization. Disturbance in phase separation and its dynamic nature contributes to formation of toxic protein aggregates in neurodegenerative disease, and could be a useful drug target in such disorders.

## Introduction

Over 20% of eukaryotic proteomes consist of polypeptide sequences that are of low complexity (LC)^1–5^. Instead of using a balanced distribution of the 20 amino acids typically deployed to facilitate the folding of a protein into a distinct, three-dimensional shape, LC domains are often composed of a limited number of amino acids^6^. A major group of proteins carrying LC domains includes the RNA- and DNA-binding proteins used in gene regulation^7^. Many of these proteins function as dynamic ribonucleoprotein (RNP) complexes in membraneless organelles, and it has long been recognized that LC domains are present in the major constituents and promoters of membraneless organelle assembly^8,9^. Other examples of LC domain-containing proteins include intermediate filaments, proteins of the central channel of nuclear pores, and the membrane-bound proteins of the Golgi apparatus and mitochondria^10–14^. Simply put, LC domains are deployed liberally in all aspects of cell biology.

**Fig. 1:**
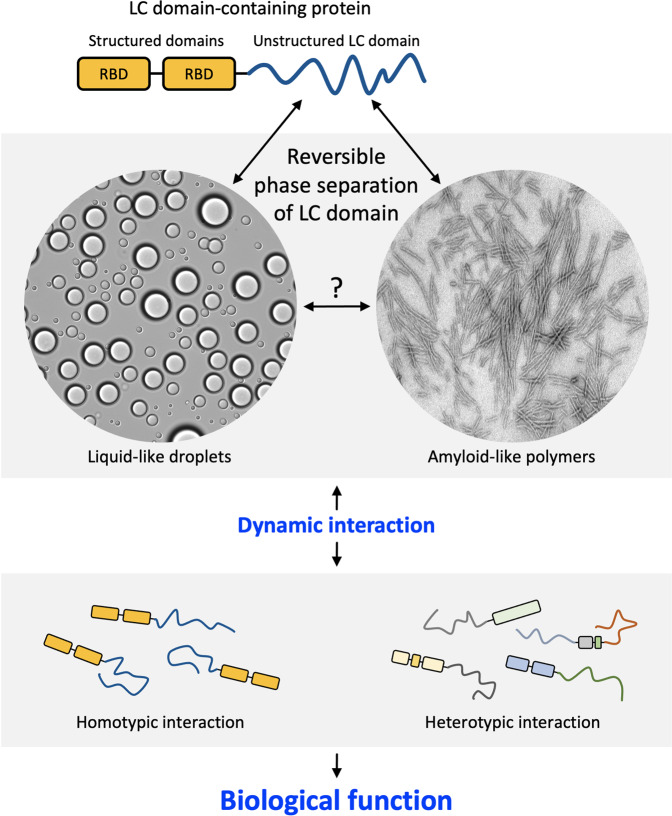
Phase separation of low-complexity (LC) domains in cellular function. Many LC domain-containing proteins consist of structured domains, such as the RBD (RNA binding domain, rectangles) and LC domain (dark blue lines). While previously thought to function in the absence of structural order, LC domains undergo reversible phase separation into liquid-like droplets and/or amyloid-like polymers. By mediating homotypic or heterotypic protein‒protein interactions, phase separation can regulate the functions of LC domain-containing proteins in diverse biological processes. The dynamic and reversible nature of phase separation is key to its biological validity and functional utility.

LC domains have long been thought to function in the absence of a molecular order and thus are termed intrinsically disordered. A decade ago, however, it was observed that these LC domains can undergo phase separation out of aqueous solution to form either liquid-like droplets (LLDs) or amyloid-like reversible polymers upon in vitro incubation^15,16^ (Fig. 1). A large body of research has revealed that phase separation provides a general strategy for the formation of membraneless organelles such as nucleoli, stress granules, and Cajal bodies by mediating reversible and dynamic protein–protein or protein–RNA interactions^8,9^. Moreover, phase separation-based interactions can contribute to the formation of a dynamic complex of proteins and RNA in a variety of cellular processes, including gene regulation, DNA repair, cell fate decisions, and even immune responses^17–19^. Consistently, disturbances in the phase separation process can cause disease. Cancer-related proteins can drive aberrant gene transcription and tumorigenesis through unbridled phase separation of the LC domains^20,21^. Amyotrophic lateral sclerosis (ALS)- or frontotemporal dementia (FTD)-causing mutations impair the dynamics and reversibility of phase separation, leading to disturbances in the dynamics and function of the RNP complex^22,23^.

Phase separation is now recognized as a fundamental principle in cellular organization. It provides the molecular basis of the dynamics of intracellular spatial organization that cannot be readily explained by traditional lock-and-key models of protein‒protein interactions. If we are to properly understand the role of phase separation in disease, we must learn how phase separation-mediated interactions actually work in biological reactions and processes. In this review, we discuss phase separation, highlight the regulatory mechanisms of phase separation, and describe how studies of model systems have revealed a role for controlled and reversible phase separation in cellular function and diseases.

## Phase separation of the LC domains

### LC domains known to undergo phase separation

How can we demonstrate that a specific protein is capable of phase separation? The most common way to initially detect phase separation is light microscopy^24,25^. In this technique, a solution of protein samples is first observed under conditions in which there are no droplets. Then, by making a change in conditions to favor phase separation (e.g., by adding or diluting salt, changing the pH or temperature, or adding RNA) followed by incubation of the sample for a fixed amount of time (minutes or hours), the solution can be imaged microscopically, and the presence of droplets can be identified. Upon prolonged incubation, the LC domains can also transition into hydrogels composed of amyloid-like polymers. The gelation of LC domain polymers has been adapted into confocal microscopic assays, wherein small hydrogel droplets are formed on the wells of chamber slides^26^. The polymeric fibers can be further imaged using fluorescence or electron microscopy^24,26,27^.

**Fig. 2:**
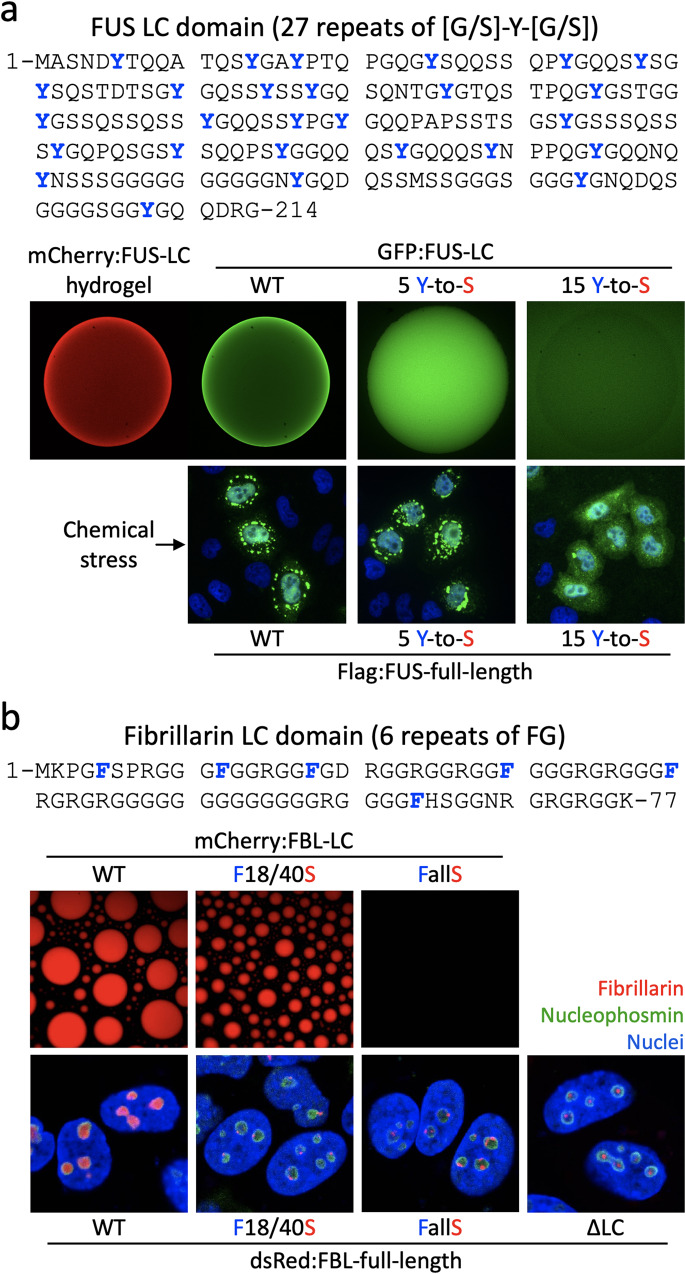
The LC domain mutations in FUS and fibrillarin correlatively affect phase separation and recruitment to membraneless organelles. **a** Mutation of tyrosine residues in the FUS LC domain impairs both hydrogel binding and stress granule association. The [G/S]-Y-[G/S] triplet repeats in the FUS LC domain are shown at the top. When the GFP-linked WT or mutant LC domains harboring 5 or 15 Y-to-S mutations are assayed for binding to mCherry hydrogel droplets composed of the FUS LC domain (mCherry:FUS-LC), moderate retention is observed for the 5 Y-to-S mutant, and little retention is observed for the 15 Y-to-S mutant. As FUS is a major constituent of stress granules, WT FUS localizes to stress granules in human U2OS cells upon application of sodium arsenite. Predictably, the 5 Y-to-S mutant localizes to stress granules, but the 15 Y-to-S mutant does not. Cell nuclei were visualized with DAPI. **b** Mutation of phenylalanine residues in the fibrillarin LC domain impaired both LLD formation and nucleolar localization. The 6 FG repeats in the fibrillarin LC domain are shown at the top. In the LLD formation assay, droplets formed by the F18/40 S LC domain are smaller in size than those made by the WT LC domain, and no droplets are assembled by the FallS LC domain, in which all 6 phenylalanine residues are substituted with serine. These mutations have correlative effects on the ability of fibrillarin to localize to the nucleolus in living cells. WT fibrillarin is evenly localized to the nucleolar dense fibrillar component region, but F18/40 S and FallS fibrillarin show aberrant, dot-like expression patterns similar to those observed with fibrillarin without the LC domain (ΔLC) (red signal). Nucleoli and nuclei are visualized with anti-nucleophosmin (green) or DAPI (blue), respectively. Panel a reproduced from Kato et al.^16^; Panel b reproduced from Kim et al.^25^.

Many LC domains that undergo phase separation contain repeats of the aromatic amino acids tyrosine or phenylalanine^10^. Tyrosine residues are usually flanked by either glycine or serine residues (G/S-Y-G/S), while phenylalanine residues have a glycine residue on their amino or carboxyl sides (FG motifs). Mutational analysis has revealed that repetitive tyrosine or phenylalanine residues are critical to the phase separation and function of LC domains. For example, tyrosine-to-serine (Y-to-S) substitutions in a triplet sequence (G/S-Y-G/S) within the LC domain of FUS or TAF15 effectively impede phase transition into the hydrogel-like state composed of polymers^21^ (Fig. 2a). In the case of the key nucleolar protein fibrillarin, we found that phenylalanine-to-serine (F-to-S) substitutions of the FG repeats are sufficient to interrupt the phase transition of fibrillarin into LLDs or hydrogel droplets and to prevent the incorporation of fibrillarin into hydrogels or LLDs composed of wild-type LC domains^25^ (Fig. 2b). Similarly, an F-to-S substitution in the LC domain of heterogeneous nuclear ribonucleoprotein A2 (hnRNPA2) can interrupt the incorporation of the LC domain into preexisting LLDs or hydrogel droplets^28^. In addition, studies of the LAF-1 P-granule protein revealed that the arginine/glycine-rich (RGG) domain plays a key role in driving phase separation^29^. Despite years of research, however, the molecular code that drives phase separation continues to be enigmatic^30^. Cross-β, π:π, and cation:π interactions have been proposed to be important drivers of reversible phase separation^10,31^, but many key questions regarding the chemical basis of phase separation remain unanswered. Further research using mutations in the LC domains will eventually yield knowledge to enable prediction of the properties of phase separation based on amino acid sequences.

### Control of reversible phase separation

Phase separation into LLDs and polymers is both dynamic and reversible (Fig. 1). Although morphologically indistinguishable from the irreversible pathogenic amyloid fibers, phase separation-driven polymeric fibers are readily labile to disassembly upon dilution, as assayed by semidenaturing detergent agarose gel electrophoresis (SDD-AGE)^6^. Pathogenic amyloid polymers remain intact and migrate through electrophoresis gels as large polymers, but even upon simple dilution, phase separation-driven polymeric fibers dissolve into gel-loading buffer and migrate in the monomeric state into SDD-AGE gels. Another test involves the application of aliphatic alcohols to determine whether the phase separation of the LC domain is dynamic and reversible. The McKnight Laboratory demonstrated the capacity of 1,6-hexanediol (1,6-HD) to melt LLDs, polymers, and intracellular organelles known to be enriched in LC domain-containing proteins, including RNA granules, nuclear speckles, and Cajal bodies^6^. As discussed below, the dynamic nature of phase separation seems to be key to its biological relevance.

Substantial evidence suggests that the control of reversible phase separation occurs through posttranslational modifications (PTMs). A variety of PTMs can alter the charge, hydrophobicity, size, and structure of LC domains. Phosphorylation is the most common PTM^32^, and phosphorylation by kinases and dephosphorylation by phosphatases provide a major control mechanism for many fundamental processes in eukaryotic cells. The phosphoryl group is negatively charged, so its addition changes a polar, uncharged residue into a negatively charged one. Depending on the protein context, the phosphate modification of an amino acid can either favor or disfavor phase separation. FUS is an example of serine/threonine phosphorylation that disrupts phase separation. The McKnight and the Tycko groups mapped 14 sites of DNA-dependent protein kinase (DNAPK)-mediated phosphorylation in the LC domain of FUS proteins and mutated pairs of serine and threonine residues to alanine as a means of blocking phosphorylation at specific sites. They found that phosphorylation by DNAPK disrupts the hydrogel binding of FUS LC domains^16^ and melts droplets of the FUS LC domain^33^. One possible explanation for this is that the negative charges introduced by phosphorylation may exert repulsive forces that reduce polymer stability^6^. The inhibition of phase separation by phosphorylation has been further documented for the head domains of intermediate filaments^14^. All 73 human intermediate filament proteins share a common domain structure consisting of a central α-helical rod domain flanked by an N-terminal head and a C-terminal tail, which are both LC domains. The head domains of the neurofilament light (NFL) and desmin intermediate filaments undergo phase separation, and this phase separation is required for assembly into mature intermediate filaments^13^. The McKnight and the Tycko groups have also shown that the PKA-mediated phosphorylation of the head domains of the NFL and desmin intermediate filaments released the head domains from the hydrogels^14^. This is in line with the known role of phosphorylation in promoting filament disassembly^34,35^.

Phosphorylation can promote the phase separation potential of certain proteins. Fragile X mental retirement protein (FMRP) is an RNA-binding protein found in neuronal granules and is involved in many biological processes, such as pre-mRNA processing^36^, translational regulation^37^, neural granule transport^38^, and ion channel binding^39^. Forman-Kay and colleagues found that phosphorylation in the LC domain of FMRP by casein kinase II promotes phase separation in vitro^40^. Similar results were observed for the microtubule-binding protein tau. Phosphorylation displayed facilitative effects for the phase separation of both tau K18 segments^41^ and full-length tau^42^. This effect was more obvious as the number of phosphorylation sites increased. Phosphomimetic mutants (serine and threonine mutated into glutamate) can only simulate this change to a limited extent, suggesting that, in addition to introducing negative charges, phosphorylation could also change the conformation of tau^42^.

Methionine oxidation provides an additional means of regulating LC domain phase separation. Yeast ataxin-2, also known as Pbp1, senses the activity state of mitochondria and is critical for autophagy upon changes in the supply of growth nutrients^43^. Kato et al. showed that the methionines of ataxin-2 LC domains can be oxidized both in vitro and in vivo and that oxidation leads to the melting of ataxin-2 LLDs^27^. Conversely, the H_2_O_2_-mediated melting of LLDs is reversed through the re-reduction of oxidized methionines via the coupled reactions of two methionine sulfoxide reductases, thioredoxin, thioredoxin reductase, and NADPH. A follow-up study showed that phase separation of TDP43 is also regulated by methionine oxidation, which implies that methionine residues might endow ataxin-2 and TDP43 with the capacity to sense the cellular redox state^43,44^.

Related to the important role of RGG domains in phase separation, PTMs of arginine residues are also important regulators of phase separation. For example, the arginine methylation of FUS reduces phase separation and stress granule association^45^. Conversely, FUS hypomethylation, a molecular phenotype of FUS inclusions in FTD, drives FUS gelation to more stable cross-β structures^46^. In addition to phosphorylation, oxidation, and methylation, a number of other PTMs have been cataloged and implicated in tuning phase separation^47–52^. Future structural studies may allow us to understand how PTMs impact intermolecular interactions and hence tune the phase separation behavior.

## Phase separation in cellular function

The biological functions of a protein depend on its physical interaction with other molecules. By mediating reversible and dynamic protein–protein interactions, phase separation can regulate the function of LC domain-containing proteins. Phase separation-dependent interactions can be achieved via two modes: homotypic interactions between the same LC domain and heterotypic interactions between different LC domains (Fig. 1). In the following, we provide two archetypes of the functional repertoire of phase separation; however, evidence for a variety of possible functions of phase separation in cells is still being acquired, and it is necessary to identify the full functions and roles of phase separation.

### Phase separation is a mechanism for membraneless organelle assembly

Inside eukaryotic cells, macromolecules are partitioned into membrane-bound compartments, including the nucleus, lysosomes, endoplasmic reticulum, chloroplast, mitochondria, and Golgi apparatus. Their membranes create discrete chemical environments and achieve separation of constituents from the bulk cytoplasm. Enclosing membrane-bound compartments requires dedicated machinery to construct and maintain the lipid bilayer and transport substances across the membrane^53,54^. Many other well-known intracellular structures, including the nucleolus, Cajal bodies, nuclear speckles, paraspeckles, stress granules, and P granules, lack membranes^55–57^, introducing the potential for greater dynamics. These membraneless organelles rapidly exchange components with the cellular milieu, and their properties are readily altered in response to environmental cues, often implicating membraneless organelles in response to stress signaling. However, the mechanistic principles of their assembly and disassembly remain unclear.

Phase separation is an appealing answer. In 2009, a study of P granules (RNA and protein-containing bodies in nematode embryos) showed that they exhibit liquid-like behavior in vivo and that LAF-1, a DDX3 RNA helicase found in P granules, phase separates into P granule-like droplets in vitro. That study demonstrated that RNAi knockdown of LAF-1 results in the dissolution of P granules in the early embryo, suggesting that LAF-1 droplets are important for P granule assembly^15^.

In 2012, McKnight and colleagues demonstrated that the components of stress granules—namely, the LC domains of FUS and hnRNPA2 RNA-binding proteins—can reversibly phase separate into polymeric and amyloid-like fibers, and this reversible transition can mediate their dynamic movement in and out of stress granules^16^. A stress granule is a cytoplasmic membraneless organelle that forms in response to a variety of cellular stressors and signaling and promotes cell survival by condensing translationally stalled mRNAs, ribosomal components, translation initiation factors, and RNA-binding proteins. The researchers showed that mCherry:FUS and mCherry:hnRNPA2 hydrogel droplets are capable of trapping the LC domains of heterologous RNA-binding proteins found in stress granules. Y-to-S mutations in G/S-Y-G/S triplet motifs of the FUS LC domain that abolish phase separation have correlative effects on the ability of FUS to be incorporated into stress granules in living cells (Fig. 2a). Furthermore, phosphorylation by DNAPK interferes with the phase separation of the FUS LC domain, explaining the dynamic translocation of FUS upon DNAPK signaling^33^. Together, these findings suggest that reversible phase separation can drive the inclusion and exclusion of RNA-binding proteins in stress granules in a way that can be regulated by the local concentration of RNA-binding proteins and PTMs, such as phosphorylation. Subsequent studies demonstrated that the phase separation of LC domains in a variety of different RNA-binding proteins—including TDP-43, TIA1, Lsm, RBM14, nucleophosmin, and fibrillarin^25,58–65^—can contribute to the assembly of stress granules, P bodies, paraspeckles, Cajal bodies, and nucleoli. For example, phase separation of the N-terminal LC domain in fibrillarin regulates its binding to RNA-binding proteins and proper nucleolus localization^25^. The nucleolus has a multilayer organization, which has been proposed to underlie the sequential assembly of ribosomal subunits^66^. Notably, LC hydrogels of fibrillarin can trap not only the same LC domains via homotypic protein–protein interactions but also heterotypic LC domains derived from RNA-binding proteins other than fibrillarin. Mutational analysis demonstrates that F-to-S mutations in FG repeats of the fibrillarin LC domain that abolish phase separation prevent both the interaction of fibrillarin with RNA-binding proteins and the normal localization of fibrillarin into dense fibrillar components within the nucleolus (Fig. 2b).

The notion that phase separation drives the dynamic assembly of membraneless organelles is further supported by the observation that the disease-causing mutations in the LC domain of RNA-binding proteins not only reduce the dynamics of the respective LC domain polymers but also reduce the RNP granule dynamics and functions in cells. For the RNA-binding proteins hnRNPA1, hnRNPA2, and hnRNPDL, D-to-V mutations in the LC domain have been identified in patients with ALS and limb-girdle muscular dystrophy^67,68^. The Taylor group found that the D-to-V mutations in hnRNPA1 and hnRNPA2 alter the dynamics of stress granule assembly in cells^58,59,68^. The McKnight Laboratory discovered a possible reason for this: the D-to-V mutations in all three proteins, hnRNPA1, hnRNPA2, and hnRNPDL, cause the respective LC domains to phase-separate into labile polymers with enhanced stability, as measured using SDD-AGE^13^. Other studies of TDP-43^62^, TIA1^63^, and FUS^60,61^ have consistently shown that disease mutations result in a propensity for more stable polymers, affecting phase separation-based interactions and slowing the assembly and disassembly of the membraneless organelles where they reside. These observations may indicate that phase separation represents the underlying biological utility of LC domains, allowing proteins to dynamically move into and out of subcellular compartments that are not membrane bound.

### Phase separation as a regulatory mechanism of gene expression

**Fig. 3:**
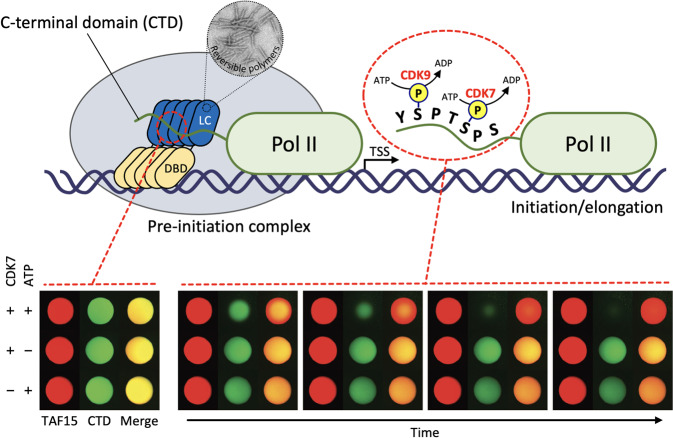
Phase separation as a regulatory mechanism of dynamic RNA polymerase II recruitment during the transcription cycle. Upper panel, schematic of RNA polymerase II recruitment by LC domain polymers. During transcription initiation, the DNA-binding domain (DBD) of FET fusion proteins binds to their cognate genes, and their LC domains form reversible polymers that can recruit RNA polymerase II (Pol II) via direct interaction with the C-terminal domain (CTD). Phosphorylation of the CTD heptad repeats by CDK7 or CDK9 facilitates release of the LC domain polymer-bound Pol II so that it can escape the preinitiation complex and enter the process of transcriptional initiation and elongation. Lower panel, hydrogel binding assay showing phosphorylation-regulated binding of GFP-linked heptad repeats 27–52 of Pol II CTD (GFP-CTD_C26_) to mCherry-TAF15 hydrogel droplets. Bound protein is exposed to both CDK7 protein kinase and ATP (top), CDK7 alone (middle), or ATP alone (bottom). Bound GFP-CTD_C26_ is released from mCherry-TAF15 hydrogel droplets in a time-dependent manner only in the presence of both CDK7 and ATP. Figure adapted from Kwon et al.^21^.

Phase separation also contributes to gene regulation, possibly by promoting the dynamic assembly of transcription factors and RNA-binding proteins. Studies of the RNA-binding proteins FUS, EWS and TAF15, offered an early example of the association between phase separation and transcription initiation machinery. These three RNA-binding proteins are referred to as FET (FUS/EWS/TAF15) proteins^69^. The translocation of the N-terminal LC domains of FET proteins to any of a number of different DNA-binding domains (DBDs) represents an oncogenic event leading to many forms of cancer^70–74^. DBDs direct the cancer-causing fusion proteins to the appropriate genes to facilitate cell growth or survival, while the N-terminal LC domains of FET proteins function as transcriptional activation domains. Despite a concrete understanding of how DBDs function, however, the mechanisms behind the function of the activation domains remain unknown. McKnight and colleagues have provided compelling evidence that the LC domains of FET proteins directly recruit the C-terminal domain (CTD) of RNA polymerase II and that the molecular determinant for this interaction is a phase separation of the N-terminal LC domains of FET proteins^21^ (Fig. 3). The CTD of mammalian RNA polymerase II contains 52 repeats of the heptad sequence YSPTSPS^75,76^. The CTD, which is 350 residues in length, is composed almost exclusively of just four amino acids—Y, S, P, and T—corresponding to the LC sequence. In vitro binding assays revealed that the CTD of RNA polymerase II is trapped by hydrogel droplets formed from the LC domains of FUS, EWS and TAF15. Y-to-S mutations in the G/S-Y-G/S triplet repeats in the N-terminal LC domain of TAF15, which abrogate phase separation capacity, correlatively reduce both CTD interaction and the transcriptional activation capacity in cells. Furthermore, CTD binding to FET protein hydrogels is reversed upon phosphorylation of the CTD by cyclin-dependent kinases (CDK7 or CDK9), which are known to phosphorylate the CTD in living cells^21^ (Fig. 3). These observations not only suggest how FET fusion drives oncogenic gene expression but also answer the question of how RNA polymerase II is recruited to the transcription initiation complex of gene promoters. However, the role of intact FUS or TAF15 proteins under normal conditions has yet to be established.

In addition to transcription initiation, a phase separation model can explain the mechanism underlying the transition of RNA polymerase II from an initiation complex to an elongation complex (Fig. 3)^21,77,78^. During the transcription cycle (initiation, elongation, and termination), RNA polymerase II is recruited to active genes in its unphosphorylated state and released for elongation following the phosphorylation of the CTD; this model assumes that both the transcription-initiation machinery and the splicing machinery can form phase-separated condensates. The transcription-initiation machinery consists of mediators, transcription factors, coactivators, and nonphosphorylated RNA polymerase II; the splicing machinery is a transient elongation condensate 50–100 bp downstream of the initiation site and consists of phosphorylated RNA polymerase II, nascent RNA, elongation factors, RNA processing factors, and specific elongation coactivators. The hypophosphorylated CTD of RNA polymerase II is preferentially incorporated into phase-separated initiation condensate, while the hyperphosphorylated CTD is preferentially incorporated into condensates that are formed by splicing factors. When RNA polymerase II reaches the end of the gene, hypophosphorylated CTD is released and transferred back to the initiation condensate.

**Fig. 4:**
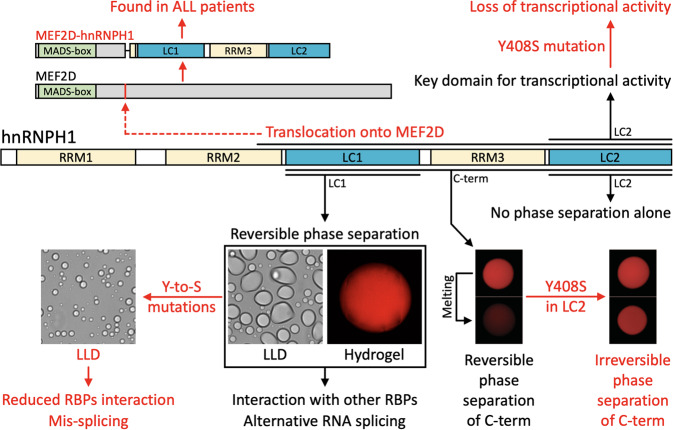
Changes in phase separation properties impact the function of hnRNPH1 in splicing and transcription. The domain architecture of hnRNPH1 shows that the N-terminal half consists of two RNA-recognition motifs (RRM1 and RRM2) and the C-terminal half consists of two LC domains (LC1 and LC2) separated by RRM3. RRMs are in yellow, and LC domains are in blue. The oncogenic MEF2D-hnRNPH1 fusion protein is shown on the top, in which hNRNPH1 maintained two LC domains and one RRM at the C-terminus and MEF2D maintained the critical MADS-box domain at the N-terminus. Out of the two LC domains of hnRNPH1, LC1 can undergo phase separation into LLDs or hydrogel droplets composed of reversible polymers. Y-to-S mutations impairing the LLD formation of hnRNPH1 LC1 domains significantly reduced interaction with different kinds of RNA-binding proteins and the alternative-splicing activity of hnRNPH1, providing insight into the role of LC1 phase separation in the control of hnRNPH1 splicing activity (bottom left). While the LC2 domain does not undergo phase separation by itself, it is required for transcriptional activation in the context of a fusion gene with the GAL4 DNA binding domain. A single tyrosine-to-serine substitution (Y408S) in the LC2 domain is sufficient to interrupt transcriptional activity. Unlike the mCherry hydrogel droplets composed of the WT C-terminal half of hnRNPH1, which were melted substantially by 1,6-HD, hydrogel droplets composed of the C-terminus containing the Y408S mutation were resistant to 1,6-HD even after overnight exposure at 37 °C, implying the importance of phase separation dynamics in hnRNPH1 functions (bottom right). Images were reproduced from Kim et al.^24^.

The role of phase separation in RNA splicing is further supported by studies of hnRNPs. hnRNPs are a large family of RNA-binding proteins that control key events in RNA biogenesis under both normal and diseased cellular conditions. Blencowe and colleagues showed that phase separation-dependent interactions control the assembly of hnRNPA/D family members, which, in turn, function to regulate splicing^79^. hnRNP complexes also contain different kinds of RNAs and RNA-binding proteins other than hnRNPs, and their assembly and disassembly occur rapidly^80^. hnRNPH1 is a prototypical hnRNP containing two distinctive LC domains on its C-terminus (LC1 and LC2). Using mutagenesis of these LC domains of hnRNPH1, we showed that a triple tyrosine substitution reduced the ability of the hnRNPH1 LC1 domain to phase separate and simultaneously reduced the capacity of hnRNPH1 to interact with RNA-binding proteins and regulate RNA splicing in living cells (Fig. 4)^24^. These results suggest that phase separation of the LC1 domain can promote the higher-order assembly of hnRNPH1 and other RNA-binding proteins that are required for the splicing activity of hnRNPH1. Thus, we speculate that phase separation-mediated assemblies relay the passage of genetic information from one site to another within a cell, ensuring that the process is of extreme fidelity.

## Phase separation in disease

Evidence accumulated over many years in studies of genetics, cell biology, and pathology has revealed that phase separation is relevant to numerous human pathological conditions, including cancer and neurodegenerative diseases. Therapeutic strategies to regulate phase separation dynamics in cells could help treat diseases related to aberrant phase separation.

### Role of phase separation in cancer

Cancer is a disease where cells reproduce uncontrollably. It is governed by biochemical pathways that have escaped the regulatory bounds of normal homeostatic balance. This balance is maintained through precise spatiotemporal regulation of these pathways. Phase separation is increasingly implicated as a previously hidden driver of aberrant spatiotemporal organization and protein dynamics involved in oncogenic activity. As discussed above, FET fusion oncoproteins provide a striking example of how the phase separation of oncoproteins acts to assemble transcription machinery and causes aberrant transcription^21^ (Fig. 3). When appended to a DBD, as is the case in oncogenic FET fusion proteins, the LC domains of FET proteins act to directly recruit the CTD of RNA polymerase II. To achieve this task, the LC domains of FET proteins must be capable of phase separation. Together with the fact that FET proteins in their intact form are endowed with an RNA binding domain and are not associated with cancer development, McKnight and colleagues proposed that the binding of multiple copies of the DBDs from fusion oncoproteins to their cognate genes may concentrate the FET LC domain to a level sufficient for phase separation, driving aberrant transcription^6^. This is in accordance with previous studies showing that portions of EWS, FUS, and TAF15 are functionally interchangeable in FET fusion proteins, while the transcription factor DNA-binding moiety determines the tumor phenotype^81^.

Consistent with the notion that the dynamic aspect of phase separation is the key to biological validity and the functional utility of phase separation, changes in phase separation dynamics functionally impact gene transcription and biochemical outcomes in cancer. One prominent example of this is the correlative effects of Y-to-S mutation of the LC domain in hnRNPH1 on phase separation dynamics and transcriptional activity in the context of fusion oncoproteins (Fig. 4). The hnRNPH1-myocyte-specific enhancer factor 2D (hnRNPH1-MEF2D) fusion gene has been identified in acute lymphoblastic leukemia. The resulting gene product is a fusion protein in which a DBD of MEF2D is connected to the C-terminal region of hnRNPH1, retaining the LC1 and LC2 domains. We have demonstrated that the LC2 domain in the C-terminal region functions as a transcriptional activation domain^25^. Remarkably, the Y408S mutation in the LC2 domain abolished its transcriptional activity. The same LC2 mutation induced the phase separation of the hnRNPH1 C-terminus into irreversible hydrogel droplets, suggesting that the Y408S mutation may decrease the transcriptional activity of hnRNPH1 by restricting the movement and interactions of other macromolecules, such as transcription factors and coactivators. This hypothesis was supported by the observation that the Y408S mutation of the LC2 domain enhanced homotypic and heterotypic binding to the LC domains of various RNA-binding proteins, including FUS, TAF15, DHX9, hnRNPA1, hnRNPA2, and hnRNPF^25^. Similar observations have been made regarding AKAP95, which is frequently overexpressed in breast and ovarian cancers^82,83^. Mutation of all six tyrosine residues to phenylalanine (Y-to-F) in the LC domain of AKAP95 enhances phase separation propensity, renders condensates into a more solid-like state, and impairs the ability of AKAP95 to regulate RNA splicing of cancer-related targets and tumorigenesis^83^. Given that AKAP95 is dispensable in normal cell growth, its overexpression might increase the concentration of the AKAP95 LC domain to a level that is sufficient for phase separation, which in turn might mediate the oncogenic interactions of AKAP95 with other macromolecules, such as splicing modulators and RNA substrates. Collectively, it is plausible that overexpression or the translocation product causative of cancer might recruit the macromolecules necessary for the initiation of oncogenic gene expression in a phase separation-dependent manner. Accordingly, therapies that target the phase separation dynamics of oncoproteins are currently in development for cancer treatment^20^.

### Pathogenic LC domain proteins in neurodegenerative diseases

Protein aggregation is a pathological hallmark of neurodegenerative diseases, including the β-amyloid (Aβ) and tau proteins of Alzheimer’s disease, the huntingtin of Huntington’s disease, and the α-synuclein of Parkinson’s diseases^84^. Many proteins found in pathological aggregates contain intrinsic disorder/LC domains^85,86^. Clear examples of a link between neurodegenerative diseases and LC proteins have been found in studies of several ALS-associated proteins. ALS is a progressive adult-onset neurodegenerative disease characterized by the selective death of motor neurons in the brain and spinal cord^87^. Different RNA-binding proteins harboring LC domains (TDP-43^88,89^, FUS^90,91^, hnRNPA1^68,92^, hnRNPA2^68^, matrin 3^93^, TIA1^63^, hnRNPDL^67^, and annexin A11^94,95^) are associated with ALS. ALS-causing mutations in the LC domains of these RNA-binding proteins accelerate phase separation into less dynamic or irreversible fibrils that can produce the fibrillar pathology observed in patient cells^13,59–63,68^. As mentioned above, these mutations can cause disturbances in the dynamics of membraneless organelle assembly, which can impair functions with adverse consequences for multiple aspects of RNA metabolism^96^.

**Fig. 5:**
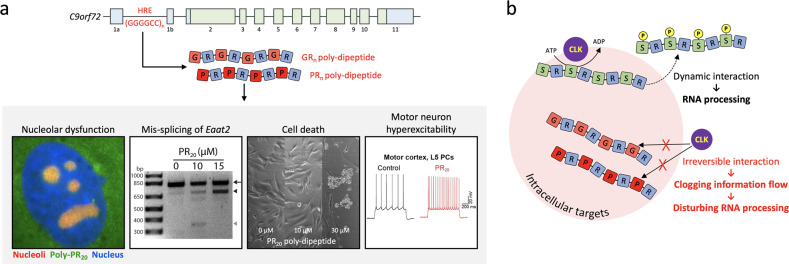
Schematic diagram showing how the soluble poly-dipeptides encoded by the *C9orf72* repeat expansion cause toxicity. **a** Cellular processes impaired by PR and GR poly-dipeptides. The *C9orf72* gene consists of 11 exons. Coding exons are indicated in green, and noncoding exons are indicated in blue (not to scale). Unaffected people contain 10-20 GGGGCC hexanucleotide repeats, but the repeat number expands to hundreds or even thousands in *C9orf72* ALS cases. A hexanucleotide repeat expansion (HRE), (GGGGCC)_n_, can produce five different poly-dipeptides, including GR and PR poly-dipeptides. Four pathogenicities enacted by GR and PR poly-dipeptides are shown: (1) PR poly-dipeptide bound to nucleoli results in nucleolar dysfunction. Nucleolar localization of synthetic peptide containing 20 repeats of the PR sequence (PR_20_) (green signal) is deduced by colocalization with the nucleolar marker fibrillin (red signal). (2) Impediments in splicing of the transcript encoding the glutamate transporter EAAT2. Human astrocytes exposed to PR_20_ show aberrantly spliced EAAT2 transcripts (black and gray arrowheads). The arrow indicates a normal EAAT2 transcript. (3) Alterations in cell morphology and death. Upon exposure to 30 µM of the PR_20_ peptide for 24 h, almost all cells were detached from the culture substrate and dead. (4) Motor neuron hyperexcitability. PR_20_ peptide application (100 nM, 20 min) causes hyperexcitability in layer 5 (L5) pyramidal cells (PCs) of the primary motor cortex (cortical motor neurons). Reproduced from Kwon et al.^23^ and Jo et al.^123^. **b** Cartoon depicting the irreversible binding of PR and GR poly-dipeptides to intracellular targets. The serine-arginine (SR) domain of SR splicing factors binds LC proteins, which is dynamically reversible by cyclin-like kinases (CLKs). PR and GR poly-dipeptides bind to LC proteins but cannot be liberated by CLK enzymes, thereby impeding the flow of information from gene to mRNA to protein.

By far, the most common genetic cause of ALS and FTD is the expansion of the GGGGCC hexanucleotide repeat (HRE) in the first intron of the chromosome 9 open reading frame 72 (*C9orf72*) gene^97–99^. From the HRE, five different poly-dipeptides are produced, depending on the reading frames, namely, glycine-alanine, glycine-proline, glycine-arginine (GR), proline-alanine, and proline-arginine (PR) poly-dipeptides (Fig. 5a)^100–106^. These poly-dipeptides are obviously LC domain proteins composed of only two amino acids. Of the five poly-dipeptides derived from *C9orf72* HRE, McKnight and colleagues recognized that PR and GR poly-dipeptides are reminiscent of the serine-arginine (SR) domains commonly found in SR splicing factors (SRSFs) that bind to hydrogels made up of the LC domain of the RNA-binding protein hnRNPA2 in vitro^23,107,108^. This SR binding to hydrogels is reversed upon the phosphorylation of serine residues by cyclin-like kinases (CLKs) known to regulate SRSF function in living cells (Fig. 5b)^109^. They thus proposed the intriguing hypothesis that soluble PR and/or GR poly-dipeptides might bind LC-domain hydrogel droplets but are impervious to CLK-mediated phosphorylation and thus irreversibly bind cellular targets such as the RNP complex in membraneless organelles in vivo. Indeed, they found that, unlike native SRSF2 protein, which is distributed across nuclear speckles, the two poly-dipeptides are irreversibly trapped in nucleoli, where they impair ribosomal RNA processing (Fig. 5a). McKnight and colleagues confirmed that the application of one of the dipeptides (PR) to cultured human astrocytes resulted in the mis-splicing of a number of gene transcripts, including the same mis-splicing of the glutamate transporter *Eaat2* transcript found in *C9orf72* ALS patients^23,110^. After this work, a number of studies produced confirmatory findings. PR and/or GR poly-dipeptides disrupt the phase separation of the nucleolar protein nucleophosmin^65^, impede nucleocytoplasmic transport^111,112^ and are themselves capable of phase separation^113,114^. Unbiased studies of the direct intracellular targets of PR poly-dipeptides showed that they bind to the polymeric forms of LC domains in a wide variety of cellular proteins, including RNA-binding proteins, nucleoporins, and intermediate filaments^13,115^. From these results, we speculate that the soluble forms of PR and GR poly-dipeptides, via irreversible binding to LC proteins, cause widespread disturbances to the integrity and dynamics of cellular structures^13,23,65,114–119^, resulting in broad impediments to the information flow from gene to mRNA to protein that eventually lead to cell death (Fig. 5a).

Our more recent study showed that PR and GR poly-dipeptides are drivers of cortical hyperexcitability, an early and key feature of ALS patients (Fig. 5a)^120–123^. Despite much research on the subject, its underlying mechanisms remain elusive. We demonstrated that PR and GR poly-dipeptides selectively induce hyperexcitability in cortical motor neurons, possibly by binding to and hyperactivating a voltage-gated sodium channel Nav1.2-β1-β4 complex. As discussed above, PR poly-dipeptides can impede splicing of the *Eaat2* transcript in astrocytes^23^. We thus speculate that this impediment might decrease the reuptake of excess glutamate that is released by hyperactivated motor neurons, favoring the accumulation of glutamate in the synaptic cleft, which, in turn, leads to glutamate excitotoxicity and eventually to the selective neurodegeneration of lower motor neurons in *C9orf72* ALS. Knowing that Nav1.2 and auxiliary β4 subunits have intrinsically disordered domains, we suggest that phase separation of these LC domains might be important for the assembly of the macromolecular sodium channel complex and interaction with other LC domain proteins, such as neuronal intermediate filaments^124–127^. If this is the case, we further hypothesize that PR and GR poly-dipeptides might bind to phase-separated LC domains otherwise adhering Nav channels to multiprotein complexes. We anticipate that the combination of both molecular and electrophysiological studies may help identify the role of intrinsically disordered domains associated with ion channel proteins in neurological and neurodegenerative disease pathophysiology, as well as in normal physiological conditions.

## Conclusions and comments

The examples of phase-separating LC domains discussed within this review illustrate how phase separation is beneficial for cells to form dynamic, multivalent protein–protein interactions. These examples also highlight the importance of regulatory mechanisms to maintain the reversibility of phase separation. The functional relevance of reversibility and the dynamics of phase separation are particularly evident when considering that these properties are directly impacted by many disease-causing mutations. Together, phase separation dynamics serve as a biological framework to explore new therapeutic approaches to devastating human diseases, including cancer and neurodegeneration.
